# A Novel DTSCR Structure with High Holding Voltage and Enhanced Current Discharge Capability for 28 nm CMOS Technology ESD Protection

**DOI:** 10.3390/mi15010096

**Published:** 2023-12-31

**Authors:** Zeen Han, Shupeng Chen, Hongxia Liu, Shulong Wang, Boyang Ma, Ruibo Chen, Xiaojun Fu

**Affiliations:** 1 Key Laboratory of Wide Band-Gap Semiconductor Materials and Devices of Education, The School of Microelectronics, Xidian University, Xi’an 710071, China; hanzeen1212@163.com (Z.H.); slwang@xidian.edu.cn (S.W.); boyangma2021@163.com (B.M.); zzu_crb@outlook.com (R.C.); 2 National Key Laboratory of Integrated Circuits and Microsystems, Chongqing 401332, China; xjfu2000@163.com

**Keywords:** electrostatic discharge (ESD) protection, DTSCR, holding voltage, current discharge

## Abstract

To cope with the much narrower ESD design window in 28 nm CMOS technology, a novel diode-triggered silicon-controlled rectifier with an extra discharge path (EDP-DTSCR) for ESD protection is proposed in this paper. Compared with the traditional DTSCR, the proposed DTSCR has an enhanced current discharge capability that is achieved by creating a slave SCR path in parallel with the master SCR path. Moreover, the improved triggering and holding characteristic can be obtained by the proposed EDP-DTSCR. By sharing the anode emitter junction, a slave SCR path is constructed that is symmetrical to the position of the master SCR path to add an additional ESD discharge path to the EDP-DTSCR. In this way, the current discharge capability of the entire device is obviously improved. The TCAD simulation result shows that the proposed device has a remarkably lower on-resistance compared with the traditional DTSCR and the DTSCR with p-type guard ring (PGR-DTSCR). In addition, it is structurally optimized to further increase the holding voltage and reduce the trigger voltage to improve the anti-latching capability of the device, which is more conducive to the ESD protection window application of 28 nm CMOS technology.

## 1. Introduction

In nano-complementary metal oxide semiconductor technology, electrostatic discharge design is becoming increasingly challenging. As semiconductor processes continue to advance, device sizes continue to shrink, oxide film thickness, junction depth and gate breakdown voltage decrease, and ESD-induced damage is becoming more severe, which makes the ESD design window much narrower [1]. Among the many ESD protection devices, silicon-controlled rectifiers (SCR) are widely used in the internal protection of CMOS integrated circuits due to their excellent robustness and low parasitic capacitance, as well as the ability to maintain higher ESD levels in a smaller area. In order to provide more effective and comprehensive on-chip ESD protection [2], researchers have invented different structures, such as lateral SCR (LSCR) [3], modified lateral SCR (MLSCR) [4], low-voltage-triggered SCR (LVTSCR) [5,6,7], and vertical SCR (VSCR) [8], which are used to improve the trigger voltage and holding voltage of SCR devices. In addition, there are some advanced auxiliary trigger circuit techniques with which to improve the conduction speed and reduce the trigger voltage of the SCR devices, such as the substrate-triggered technique [9,10], the GGNMOS-triggered technique [11], the double-triggered technique [12], the self-triggered technique [13] and so on.

In a typical 28 nm CMOS technology, the operation voltage is 1.1 V and the maximum allowable gate oxide breakdown voltage is about 5.1 V [14]. Therefore, the ESD design window after calculating the 10% safety margin should be in the range of 1.2 to 4.6 V, which is much smaller than its predecessor. That said, the existing SCR protection devices need to adjust their trigger voltage and holding voltage to avoid oxide overstress and device latch-up.

To further reduce the trigger voltage, a diode-triggered silicon-controlled rectifier (DTSCR) has been invented [15,16,17,18]. The diode-assisted triggering mechanism can effectively reduce the trigger voltage of SCR devices to achieve a narrower ESD design window for the application [19] and, in order to better meet the requirements of the new era of electronic power systems, several new schemes have been proposed. Li et al. designed a 2D fine layout DTSCR to improve the ESD robustness of the device [20]. Additionally, the Sziklai/Darlington DTSCR proved to have faster turn-on speeds and excellent I–V static characteristics [21] and the DTSCR-EMOS-ID [22] has a lower trigger voltage, smaller parasitic capacitance and faster response time. However, the drawbacks of these reported solutions, including larger R_on_, weaker current handing, limit their practical applications.

In this paper, a new symmetrical master–slave structure is proposed to cope with the current drain capability of the DTSCR device by utilizing the constructed additional slave SCR, in combination with the master SCR. It has been shown that the new structure has a lower on-resistance and high holding voltage as well as greater current drain capability compared with the traditional DTSCR and PGR-DTSCR.

## 2. Device Structure Description

**Figure 1:**
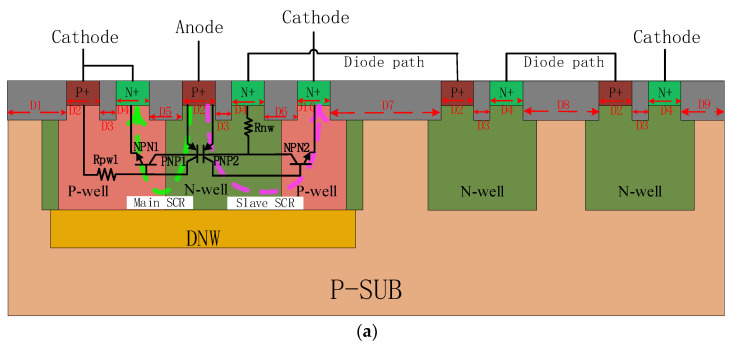

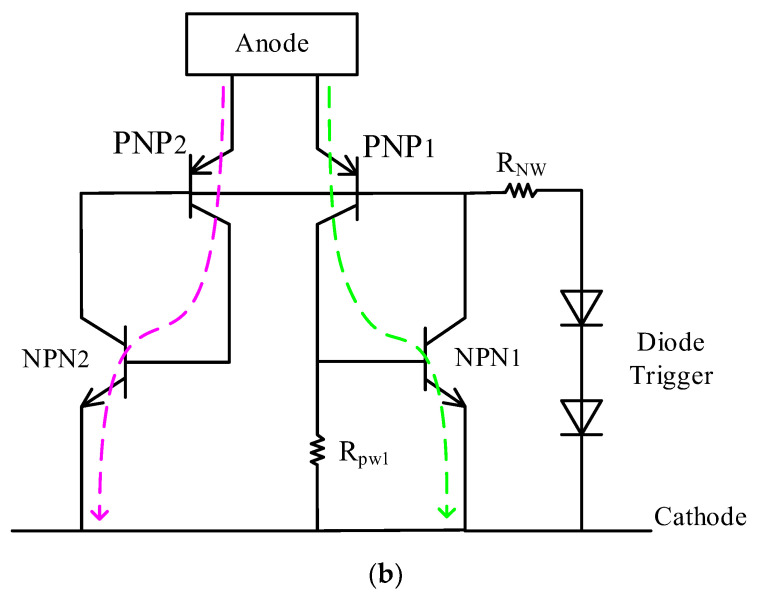
(**a**) Cross-sectional view and (**b**) equivalent circuit of the EDP-DTSCR.

In [23], a new SCR structure with double-triggering operation and that utilizes an additional high-beta parasitic n-p-n bipolar transistor, with improved current driving ability, triggering characteristics, and reverse characteristics, is proposed with design parameters for holding voltage but with limited application in low-voltage scenarios. In order to satisfy the application in 28 nm CMOS technology and improve the current drain capability in a limited area, this paper proposes a novel diode-triggered silicon-controlled rectifier structure with an extra discharge path (EDP-DTSCR) based on a traditional DTSCR. Figure 1 shows the new structure with the equivalent circuit diagram of the proposed new DTSCR device.

Based on the traditional DTSCR device, the new DTSCR device builds an additional slave SCR path to the right side of the anode by adding an N+ region symmetrical to the cathode. The new slave SCR and the master SCR together form the SCR part of the new device, and both share the same emitter junction at the anode, which means that the emitter junction of PNP1 and PNP2 are the same. The master SCR and slave SCR are also separated from the diode string section by a deep N-well to prevent interference from noise coupling. Furthermore, the new slave SCR forms an additional parallel discharge path, reducing on-resistance and increasing robustness.

During an ESD event, the diode-triggered path is turned on first; that said, the trigger voltage of the device will be controlled by the total voltage drop of the diode string. The conduction current will first pass through these diodes and conduct the slave SCR, consisting of the P+, N-well, right P-well, and N+ region, thus creating an additional conduction path and generating the first snapback (the purple dashed line in Figure 1b). As the current continues to increase and when the current of the slave SCR reaches a sufficiently large level, the master SCR will be triggered and begin to conduct the ESD-induced current (the green dashed line in Figure 1b), yielding the second snapback for ESD protection. Because of the additional parallel drain path from the slave SCR, the EDP-DTSCR has lower on-resistance and higher current drain capability than the traditional DTSCR.

**Figure 2:**
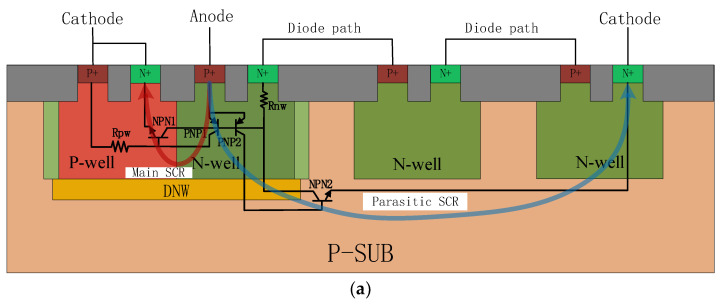

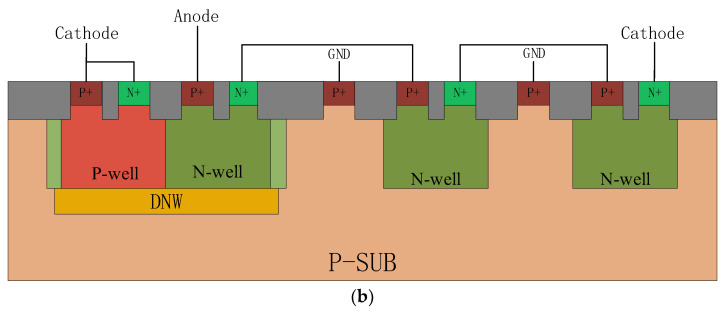
Cross-sectional views of (**a**) traditional DTSCR and (**b**) PGR-DTSCR.

The cross-sectional views of the traditional DTSCR and the DTSCR with p-type guard ring (PGR-DTSCR) are shown in the Figure 2a,b.

## 3. Results and Discussion

**Figure 3:**
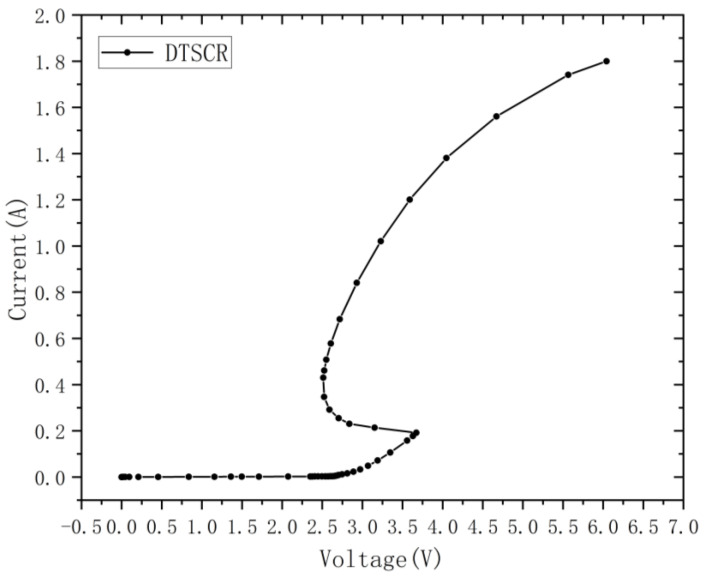
I–V characteristic curves of the DTSCR fabricated in the literature.

In order to verify the operation mechanism and key characteristics of the proposed EDP-DTSCR device, Sentaurus TCAD is used for the device simulation in this paper. In order that the simulation work in this paper be more in line with the actual device performance, before designing the device we firstly consulted a large amount of the literature. We did this in order to establish the DTSCR model and select the IV characteristic curve of the DTSCR fabricated in [24] using the 28 nm 0.9 V/1.8 V CMOS process with a device width of 50 μm. The established DTSCR model was calibrated according to its actual ESD characteristics measured with a transmission line pulse tester to obtain the initial DTSCR dimensional parameters. After calibration, the DTSCR model is able to fit the I–V characteristic curve to the actual measured data during the period between the diode triggering and the establishment of full conduction of the SCRs with positive feedback, which is valuable for evaluating the real-world performance of the DTSCRs in a 28 nm process. Figure 3 shows the I–V characteristic curve of the calibrated DTSCR.

For further improvement and optimization, on the basis of the calibrated DTSCR model, we propose an EDP-DTSCR device model with a device width of 7.9 μm. The lateral key dimensions of the EDP-DTSCR device are demonstrated in Table 1.

**Figure 4:**
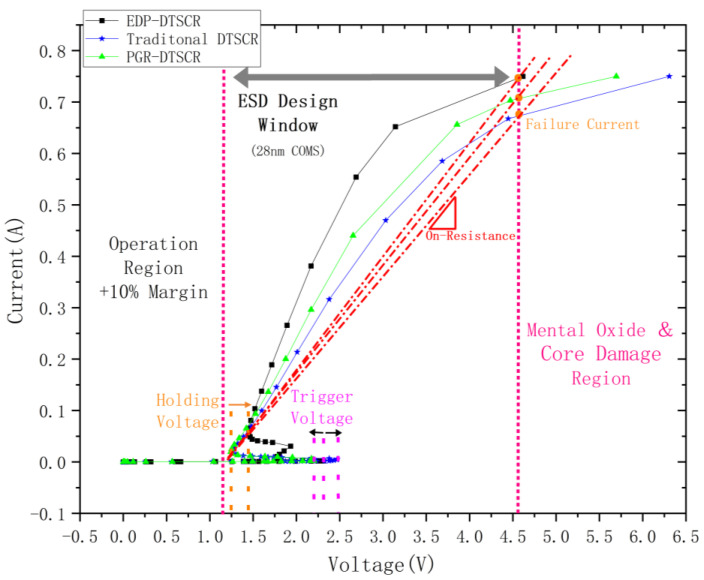
I–V characteristic curves of the traditional DTSCR, PGR-DTSCR and EDP-DTSCR.

The I–V characteristic simulation curves of the traditional DTSCR, PGR-DTSCR and the proposed EDP-DTSCR are shown in Figure 4. Among these, EDP-DTSCR shows a double snapback behavior. At the first snapback, the trigger voltage of EDP-DTSCR is 2.21 V, which is 0.17 V higher than that of the traditional DTSCR and 0.24 V lower than that of the PGR-DTSCR.

When the current increases to 26.5 mA, the master SCR conducts and yields the second snapback with the holding voltage of 1.451 V. Compared with the 1.265 V of the traditional DTSCR and the 1.243 V of the PGR-DTSCR, the holding voltage of EDP-DTSCR is improved by 0.186 V and 0.208 V, respectively. Thus, it can be seen that the ESD protection window of the EDP-DTSCR is much narrower and the scope of application is much wider.

By further analyzing the ESD protection window, the failure currents (It2) of the three devices at the window boundary of 4.6 V are 0.674 A, 0.708 A and 0.75 A, respectively. After calculation, we found the on-resistance of the traditional DTSCR to be about 4.96 Ω and the on-resistance of the PGR-DTSCR to be about 6.54 Ω, while the on-resistance of EDP-DTSCR is only 4.56 Ω. This shows that the additional parallel discharge path can effectively reduce the on-resistance of the device, improve the robustness and enhance the discharge capability.

However, note that, in Figure 3 and Figure 4, and when compared with the measured data in the literature, the I–V curve shows a small deviation after the node of i = 0.5 A, which may be due to the partial resistance increment caused by self-heating of the device during the leakage of the amplified current, as well as the convergence of the simulation model and algorithm. The actual on-resistance and robustness of EDP-DTSCR will be much better. Thus, in order to facilitate the analysis and be able to describe more accurately the internal physical mechanisms of the EDP-DTSCR, we will pay more attention to the period between the SCR triggering and the current level reaching 0.5 A in the later work.

The double snapback phenomenon demonstrated by EDP-DTSCR in Figure 4 is caused by the multiple triggering effect [25,26]. This effect causes the device to turn on slower and the trigger voltage to rise. In practice, the diode string in the DTSCR is usually placed next to the main SCR device in order to save space. This can easily cause multi-trigger effects. In Figure 2, we have labelled the two paths, the main SCR path and the parasitic SCR path, that trigger the multiple triggering effect. The parasitic SCR path consists of the P+ (anode)/N-well/P-sub and the N-well of the last diode (the blue line in Figure 2a), which dominates the current conduction due to its lower resistance than the other parasitic SCR paths.

When an ESD event occurs, the diode string opens first, and the vertically parasitic PNP1 of the main SCR conducts. At the same time, the vertically parasitic PNP2 of the parasitic thyristor begins to participate in conduction. This is because their emitter–base junction is forward biased and will result in a large number of holes being injected into the P-substrate from the anode P+ region, thus increasing the potential of the P-substrate. As the current increases, the lateral parasitic NPN2 of the parasitic SCR turns on, which in turn conducts the entire parasitic SCR. Eventually, when the main SCR reaches a certain current level, the NPN1 junction opens and the main SCR forms positive feedback and begins to conduct. Both the parasitic SCR and the main SCR have a fast return region, which is called the multiple triggering effect.

Note here that, due to the difference in current gain of the NPN transistors in the two SCRs, the parasitic SCR path will conduct first with the main SCR path. The β of NPN2 is much higher than that of NPN1, due to the fact that the emitter-to-base doping ratio of NPN2 is much greater than that of NPN1 [27].

The additional slave SCR constructed in the EDP-DTSCR device is still essentially a parasitic SCR. However, it has lower resistance and shorter current path compared with other parasitic SCRs. It should be noted that the traditional DTSCR in this paper does not suffer from the multiple triggering effect reported in [28]. This may be due to the fact that, in more advanced CMOS processes, the suppression of parasitic transistors related to the substrate will be more stringent in order to improve transistor integration, which will in turn weaken the conductivity modulation effect and thus generate a no-snapback I–V characteristic of the parasitic SCR in the traditional DTSCR. As a result, the I–V curve of a conventional DTSCR exhibits a single snapback phenomenon. This further validates the idea that the simulation work in this paper is quite reliable in terms of the advanced process of 28 nm CMOS technology.

On the other hand, for the slave SCR of EDP-DTSCR, the shorter current path will enhance the conductivity modulation effect and thus result in a snapback I–V characteristic. Therefore, the I–V curve of the EDP-DTSCR presents a double snapback phenomenon.

**Figure 5:**
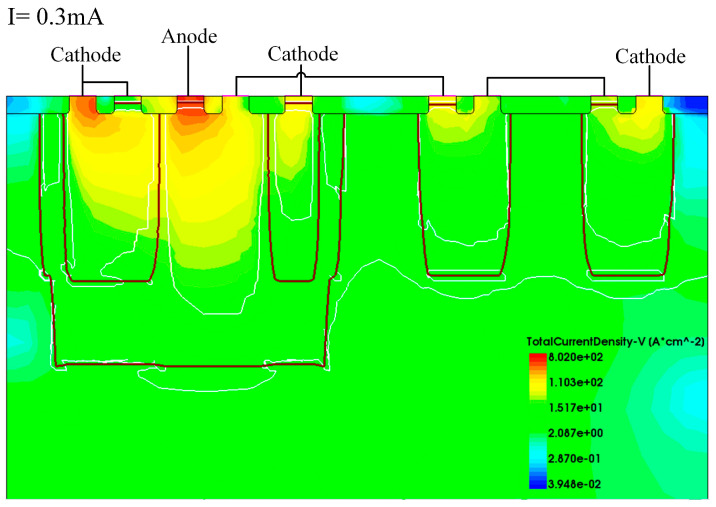
Diode-triggered path turns on.

**Figure 6:**
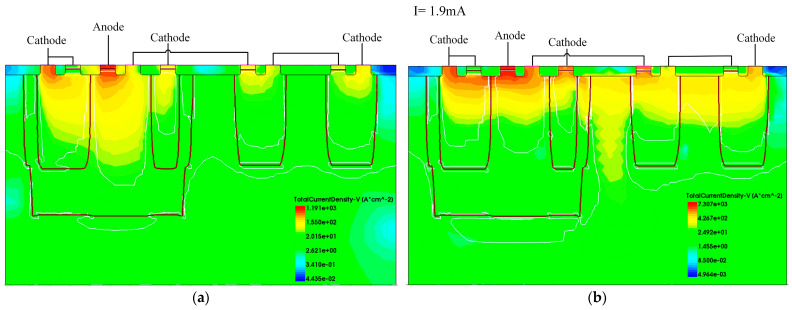
(**a**) Before and (**b**) after the slave SCR triggers.

**Figure 7:**
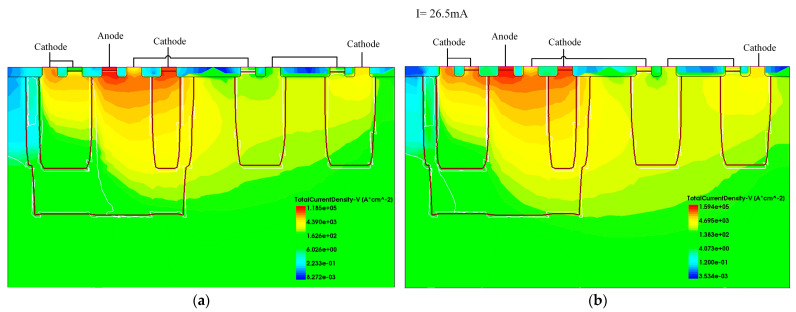
(**a**) Before and (**b**) after the master SCR triggers.

We verified the operation mechanism of EDP-DTSCR with TCAD simulation. Figure 5, Figure 6 and Figure 7 show the current density profiles of EDP-DTSCR at different moments.

It can be clearly seen in Figure 5 and Figure 6 that the diode-triggered path has already turned on at a rather small current of 0.3 mA, while the PNP1 transistor of the master SCR has also conducted. As the conduction current increases, and upon reaching 1.9 mA, the slave SCR fully conducts and begins to drain a small portion of the ESD current.

Figure 7 shows the current density profile of the proposed DTSCR before and after triggering. As the conduction current increases to 26.5 mA, the voltage drop across the left P-well reaches 0.7 V, which exceeds the built-in voltage of the PN junction. This causes the emitter junction of the NPN1 transistor to forward bias and enter an amplified state. The master SCR triggers and the conductivity modulation effect occurs, yielding the second snapback. Then, NPN1 and PNP1 transistors form a positive feedback circuit that begin to drain most of the ESD current.

**Figure 8:**
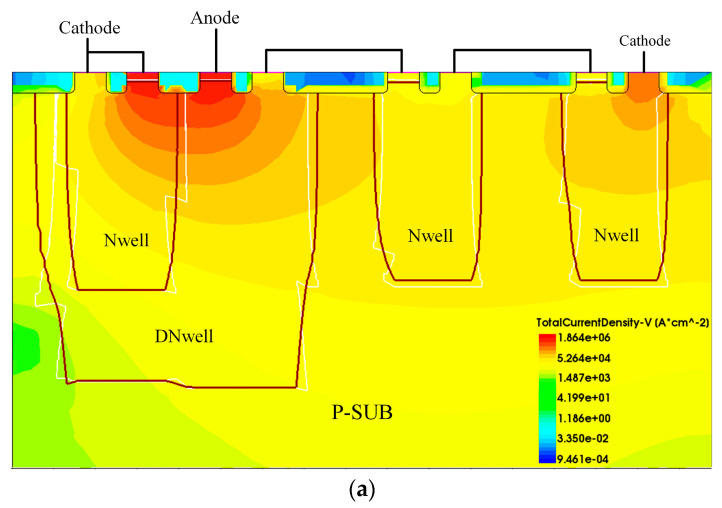

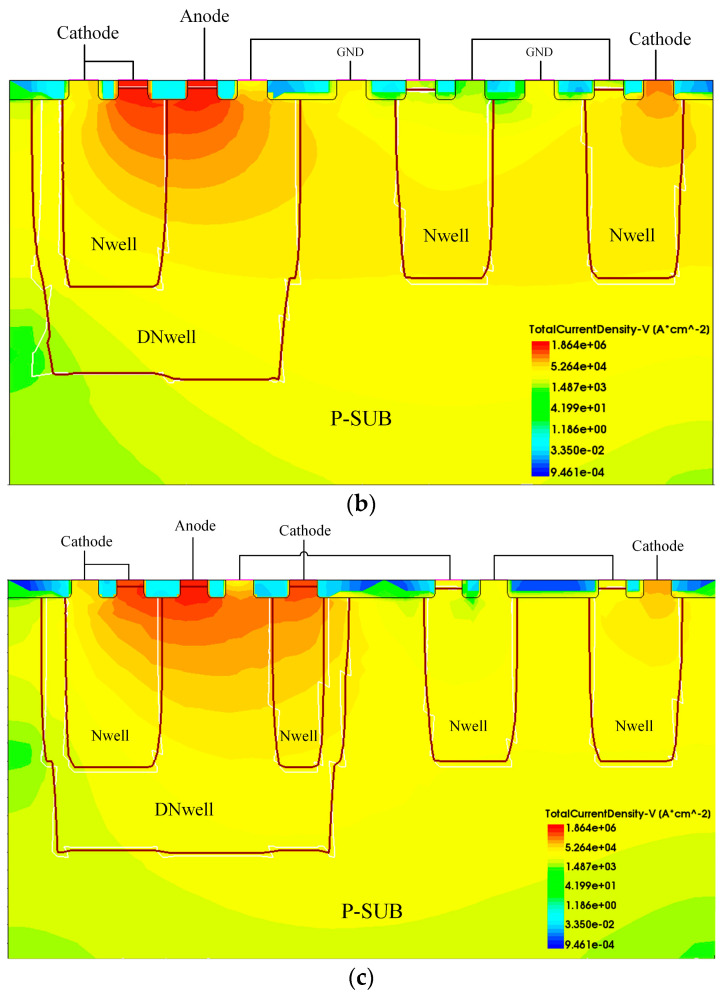
The current density profile of (**a**) the traditional DTSCR, (**b**) PGR-DTSCR and (**c**) EDP-DTSCR after full conduction.

Figure 8 shows the current density profile of the traditional DTSCR, PGR-DTSCR and the proposed DTSCR after full conduction. It can be clearly seen in the figure that the current discharge mode of all three is dominated by the SCR path. However, among these, the slave SCR structure of the proposed DTSCR gives it an additional parallel discharge path and its current discharge capability is significantly better than that of the previous two. Because the presence of a parallel drain path from the slave SCR increases the number of free electrons per unit area of the device, the device conductance increases, as evidenced by a decrease in the resistance of the EDP-DTSCR during conduction.

At the same time, the slave SCR competes with the master SCR in current distribution, and a portion of the collector current of PNP1 will be drained to the cathode through the slave SCR path, which inhibits the electron injection from the NPN1 emitter N+ to the base region of the left P-well. As a result, the emitter efficiency of the NPN1 transistor decreases, the current gain between the NPN1 and the PNP1 is reduced, the positive feedback process is slowed down, and the device’s holding voltage is increased.

## 4. Optimization of Lateral Key Characteristics

According to the above analysis, increasing the holding voltage of EDP-DTSCR requires suppressing positive feedback. Assuming that $\beta_{\mathrm{NPN}1}$ and $\beta_{\mathrm{PNP}1}$ are the current amplification coefficients of NPN1 and PNP1 in the main SCR, respectively, then the larger $\beta_{\mathrm{NPN}1}*\beta_{\mathrm{PNP}1}$ is, the larger the positive feedback effect is. The current amplification factor is expressed as:

(1) $$\beta =\frac{\alpha }{1-\alpha }$$

(2) $$\gamma =\frac{\alpha }{\beta^{*}}$$

From Equation (2) it can be seen that, when decreasing the emitter injection efficiency γ, the total base current amplification factor α decreases, and the corresponding current amplification factor β then decreases according to Equation (1). In EDP-DTSCR, the slave SCR competes with the master SCR to distribute the drain current. Reducing the resistance from the slave SCR results in more current being discharged to the cathode via the path from the slave SCR, which inhibits the injection of electrons from the emitter of NPN1 into the base region of the left P-well and reduces the emitter injection efficiency of NPN1. Thus, the resistance of the slave SCR is inversely proportional to the current gain of the master SCR, as a result, reducing the injection efficiency to further increase the holding voltage can be achieved by reducing the resistance of the slave SCR. Based on the above analysis, and keeping the other dimensional parameters of the EDP-DTSCR unchanged while adjusting D10 to change the resistance of the slave SCR path, it can be verified that changing the corresponding parameters can improve the holding voltage of the device.

**Figure 9:**
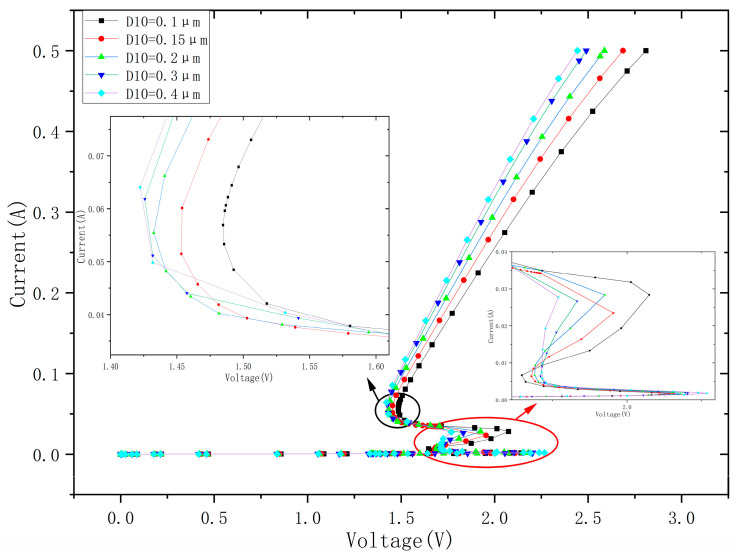
I–V characteristic curves of EDP-DTSCR at the change of D10.

Changing the lateral dimension D10, the I–V characteristic curve of EDP-DTSCR is shown in Figure 9. It can be seen that the second holding voltages are 1.422 V, 1.426 V, 1.432 V 1.453 V and 1.486 V for D10 dimensions 0.1 μm 0.15 μm, 0.2 μm, 0.3 μm and 0.4 μm, respectively. Reducing the size of the lateral dimension of D10 can reduce the resistance from the SCR, weaken the emitter injection efficiency of NPN1, decrease the current gain, lower the positive feedback, and further achieve the increase of the holding voltage.

Meanwhile, the first trigger voltages are 2.141 V, 2.157 V, 2.179 V, 2.204 V and 2.267 V for D10 dimensions 0.1 μm, 0.15 μm, 0.2 μm, 0.3 μm and 0.4 μm, respectively. As shown in Figure 9, a larger D10 can increase on-state resistance of the slave SCR and then increase the trigger voltage. Notice that all of the devices trigger at the same current level of 0.025 A because of the identical intrinsic SCR.

## 5. Conclusions

In this paper, a new ESD protection device based on the traditional DTSCR was proposed. The proposed DTSCR has a stronger current drain capability, improves holding voltages and reduces the trigger voltage to a certain extent. Its excellent ESD protection performance stems from the additional discharge path of the slave SCR to reduce the on-resistance of the device, which gives it a higher current drive capability and can help discharge the ESD current to protect the circuits more effectively.

The advantages in low-voltage ESD protection applications and the of the proposed DTSCR are verified through TCAD simulation, comparing them with traditional DTSCR and PGR-DTSCR. By further optimizing the device parameters, we can see that reducing the size of D10 can somewhat increase the holding voltage, indicating that the proposed DTSCR will be more suitable for ESD protection window in 28 nm CMOS technology.

## Figures and Tables

**Table 1:** The lateral key dimensions of the EDP-DTSCR device.

D	Length/μm
D1	0.8
D2	0.3
D3	0.2
D4	0.3
D5	0.4
D6	0.4
D7	1.3
D8	1
D9	0.5
D10	0.3

## Data Availability

Data are contained within the article.
